# Association between coronary artery disease and clinical outcome in cancer patients: A propensity score matching analysis

**DOI:** 10.1016/j.heliyon.2024.e28262

**Published:** 2024-03-16

**Authors:** Zengfa Huang, Tao Li, Shan Zhang, Bei Jiang, Mei Li, Beibei Cao, Hongfeng Zhang, Zhiqiang Zhou, Zheng Huang, Xiang Wang

**Affiliations:** a Department of Radiology, The Central Hospital of Wuhan, Tongji Medical College, Huazhong University of Science and Technology, Wuhan, 430014, China; b Department of Community Health, Wuhan Hanyang Center For Disease Prevention and Control, Wuhan, 430050, China; c Department of Pathology, The Central Hospital of Wuhan, Tongji Medical College, Huazhong University of Science and Technology, Wuhan, 430014, China; d Department of Anesthesiology and Pain Medicine, Hubei Key Laboratory of Geriatric Anesthesia and Perioperative Brain Health, and Wuhan Clinical Research Center for Geriatric Anesthesia, Tongji Hospital, Tongji Medical College, Huazhong University of Science and Technology, Wuhan, 430030, China

**Keywords:** Coronary computed tomography angiography, Coronary artery disease, Prognosis, Cancer, Propensity score matching

## Abstract

**Objective:**

The aim of this study was to evaluate the prognostic value of coronary artery disease (CAD) detected by coronary computed tomography angiography (CTA) to predict the risk of all-cause mortality in cancer patients in a propensity score matching (PSM) analysis.

**Methods:**

A total of 331 patients who previously had cancer and underwent coronary CTA from January 2015 to December 2019 were included. Multivariate Cox proportional hazards regression analysis and propensity-score matching analysis were performed. The primary endpoint was all-cause of mortality.

**Results:**

In total, 125 with CAD and 206 with no CAD during a median follow-up of 3.3 years were included in this study. After PSM, age (*HR*, 1.040; *95%CI*, 1.001–1.081; *p* = 0.014) and CAD (*HR*, 2.164; *95%CI*, 1.057–4.430; *p* = 0.035) remained significant factors for all-cause mortality.

**Conclusion:**

CAD evaluated by coronary CTA was found to be at higher risk for all-cause mortality in cancer patients. Due to the retrospective design and lack of information on some medical history and treatments, especially immune checkpoint inhibitors, a large-scale prospective study is needed to further determine the prognostic value of coronary CTA in cancer patients.

## 1 Introduction

Cardiovascular disease (CVD) and cancer are the leading causes of death in developed countries, accounting for two-thirds of disease-related mortality [1]. The 5-year disease-specific survival rates have been improved significantly in the 10 most common malignancies since the advances in early detection and treatment [2]. Nowadays, there are more than 16.7 million cancer survivors in the United States [[2], [3], [4], [5]]. For all cancers combined, 36.9% of cancer patients in China will survive at least 5 years after diagnosis around 2015 [6]. This represents a population that is vulnerable to coronary artery disease (CAD) owing to a well-established nexus between cancer and CAD.

The value of coronary computed tomography angiography (CTA) has been established and endorsed by societal guidelines as the first-line diagnostic test for patients with suspected CAD [7,8]. Using invasive coronary angiography (ICA) as the standard, the sensitivity and specificity to detect of CAD by coronary CTA was 85%–95% and 83%–90%, respectively [9,10]. Moreover, CAD identified by coronary CTA showed increased risk for all-cause death compared with no CAD [11]. However, this prognostic value of noninvasive evaluation has been reported only in suspected or confirmed CAD patients. Whether this prognostic value exists in cancer patients evaluated by coronary CTA is unclear. Thus, the aim of this study was to evaluate the prognostic value of CAD detected by coronary CTA to predict the risk of all-cause mortality in cancer patients in a propensity score matching (PSM) analysis.

## 2 Methods

This retrospective study was approved by the Institutional Review Board of the Central Hospital of Wuhan, Tongji Medical College, Huazhong University of Science and Technology. We confirmed that all methods were performed in accordance with the related guidelines and the principles of the Declaration of Helsinki. Written informed consent was waived because of its retrospective observational nature.

### 2.1 Study population

**Fig. 1:**
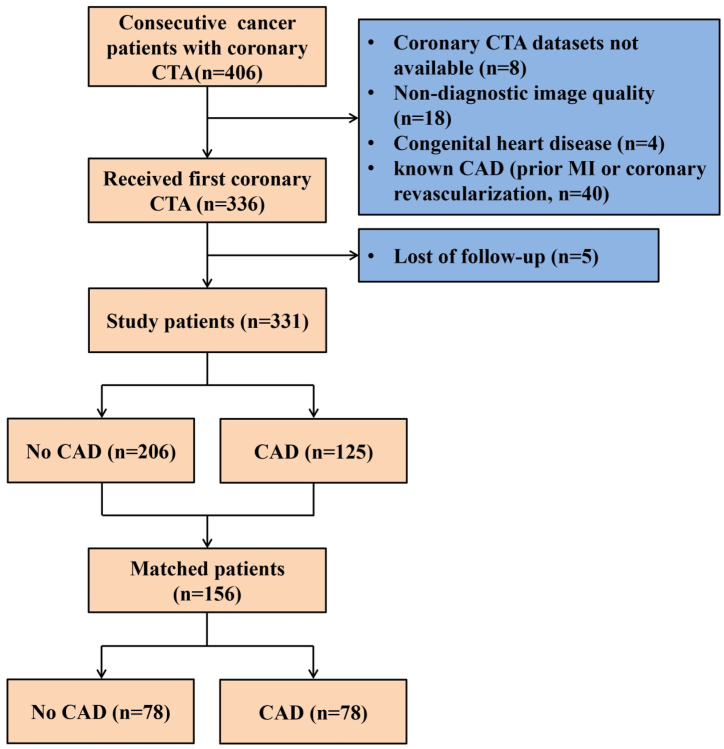
Flowchart illustrates exclusion criteria and final study population.

This study population consisted of 406 consecutive cancer patients (former diagnosis by pathology) with suspected CAD patients who underwent coronary CTA from January 2015 to December 2019 (Fig. 1) and parts of the patients have been included in our recent report [12]. Patients included in our analysis meet the following inclusion criteria: 1) adults who were 18 years old or older; 2) refer for coronary CTA using a ≥64-detector row scanner; 3) good quality images acquired that could be diagnosed with standardized reporting of segmental coronary stenosis [13]. At last, a total of 331 patients were included in the final analysis.

### 2.2 Coronary CTA protocol and image interpretation

Coronary CTA is recommended as the initial test for suspected CAD patients according to the ESC guideline [7]. The following four CT systems was used to perform coronary CTA: Aquilion 64, Toshiba Medical Systems, Otawara, Japan; Philips Brilliance iCT, Philips Medical Systems, Best, the Netherlands; uCT 760, United Imaging, China; SOMATOM Definition AS, Siemens Healthineers, Germany which has been reported previously [12]. Heart rate control (HR ≥ 65 beats/min) was performed with beta-blockers before the scan. Scanning parameters were as following: Tube voltage 120 kV, tube current 280–300 mAs. For contrast enhancement, 60–80 mL of iopromide (370mgI/mL, Bayer Schering Pharma, Germany) followed by 30–40 mL of pure saline with a flow rate of 4–5 mL/s. The iodine contrast agent was automatically triggered into descending aorta of 100 Hounsfield unit (HU) threshold units. Then the scanning was performed during an inspiratory breath hold of 8–14 s after delay of 2 s. The reconstruction images were automatic send to one of the four workstations VITREA 2 (version 6.1, Vital Images, Inc, Minnesota, America), Intelligence space portal (Version 6.0.4, Philips Medical Systems, Best, the Netherlands), UIH Advanced Workstation (uWS-CT, R004, United Imaging Healthcare, Shanghai, China), SyngoVIA (Siemens Medical Solutions, Forchheim, Germany).

Coronary CTA was performed by two independent readers using axial source images, multiplanar reformation (MRP), maximum intensity projections (MIP), curved multiplanar reformation (cMPR), and volume-rendered (VR) reconstruction images according to the guidelines of the Society of Cardiovascular Computed Tomography (SCCT) [13]. Disagreement between readers was then solved by consensus reading. Coronary artery was assessed using an 18-segment model and stenosis severity was visually assessed for each coronary plaque and categorized as: normal, absence of plaque and no luminal stenosis; minimal, plaque with <25% stenosis; mild, 25%–49% stenosis; moderate, 50%–69% stenosis; severe, 70%–99% stenosis; occluded. CAD was defined as a stenosis ≥50% in at least one coronary segment. No CAD was defined as normal or stenosis <50% in any of the coronary segments in the present study. Coronary CTA datasets were analyzed by 1 of 2 expert readers in coronary CTA who were also blinded to clinical information and outcome. Upfront interobserver reliability was assessed using 60 randomly selected coronary CTAs among the readers. The agreement between the readers was excellent (Kappa = 0.805; 95% CI: 0.641–0.968).

### 2.3 Follow-up

The primary endpoint was all-cause mortality. Follow-up procedures were approved by our hospital's institutional review board. Death status was ascertained by querying the local Community Health Service Centers. For death outside of the city, event was determined through telephone call, or review of medical records. The deadline date of follow-up was June 30, 2021.

### 2.4 Statistical analysis

Continuous variables were presented as mean ± SD. Categorical variables are presented as frequencies and percentages. We used student's t-test for continuous variables between groups. Categorical variables were compared using a chi-square test or Fisher's exact test as appropriate. PSM was adopted using 1:1 nearest neighbor matching to balance the distinction of baseline covariates [14]. Patients were matched for age, sex, BMI, history of smoking and alcoholism, hypertension, diabetes mellitus, cancer subtypes, histological grading, AJCC stage [15], ejection fraction, total cholesterol, high density lipoprotein, bold urea nitrogen and serum creatinine. Matched groups were compared with standardized differences with a 0.1 tolerance [16]. We then assessed balance before and after PSM using standardized mean differences (SMD) with the threshold of 0.20 [17]. After PSM, Univariate and Multivariate Cox proportional hazards regression models were used to calculated time to death of all cause and hazard ratios (*HR*) with 95% confidence intervals (*95% CI*) in both before PSM and PSM study. Kaplan-Meier method was used to estimate cumulative all-cause mortality, and curves were compared with the log-rank test after PSM study. We further compared 1 year, 3 year and 5 years all-cause mortality between no CAD and CAD group after PSM. A two-tailed *P* < 0.05 was considered statistically significant. All statistical analyses were performed using Stata version 16 (StataCorp LP,College Station, Texas, USA), R statistical package (version 4.0, R foundation for Statistical Computing, Vienna, Austria) and MedCalc Statistical Software version16.8.4.0 (Ostend, Belgium).

## 3 Results

### 3.1 Overall patients characteristic

In total, 331 patients (125 with CAD, 25 patients were performed PCI after coronary CTA) with a median follow-up of 3.3 years were included in this study. Patients in CAD group were older (mean age 69 vs. 63.3, *p* < 0.001), more likely male (69.6% vs. 41.7%, *p* < 0.001) with lower BMI (22.7 vs. 23.7, *p* = 0.013), higher serum creatinine level (79.6 vs. 69.5, *p* = 0.007), more likely to have smoking (37.6% vs. 11.2%, *p* < 0.001), alcoholism (22.5% vs. 5.8%, *p* < 0.001) or diabetes mellitus (28% vs. 16.2%, *p* = 0.01) compared to patients in no CAD group. There were similar findings in hypertension, total cholesterol, high density lipoprotein and blood urea nitrogen levels between CAD group and no CAD group (Table 1).

**Table 1:** Baseline characteristics before and after PSM.

Characteristic	Before PSM				After PSM			
	CAD group (n = 125)	No CAD group (n = 206)	p value	SMD	CAD group (n = 78)	No CAD group (n = 78)	p value	SMD
Age (years)	69.0 ± 8.3	63.3 ± 9.3	<0.001	0.62	68.1 ± 7.6	64.6 ± 9.4	0.098	0.20
Gender (male)	87 (69.6)	86 (41.7)	<0.001	0.54	44 (56.4)	37 (47.4)	0.336	−0.10
BMI (kg/m^2^)	22.7 ± 3.1	23.7 ± 3.8	0.013	−0.26	22.6 ± 3.0	23.1 ± 3.5	0.354	0.14
History of smoking	47 (37.6)	23 (11.2)	<0.001	0.41	14 (17.9)	10 (12.8)	0.506	−0.10
History of alcoholism	28 (22.5)	12 (5.8)	<0.001	0.28	8 (10.3)	6 (7.7)	0.780	−0.02
Hypertension	60 (48)	93 (45.1)	0.348	−0.04	38 (48.7)	38 (48.7)	1.000	0.20
Diabetes mellitus	35 (28)	34 (16.5)	0.010	0.23	17 (21.8)	13 (16.7)	0.543	−0.10
Cancer subtypes			<0.001	−0.46			0.499	0.14
Digestive system cancer	66 (52.8)	80 (38.8)			41 (52.6)	34 (43.6)		
Lung cancer	24 (19.2)	21 (10.2)			13 (16.7)	12 (15.4)		
Prostate cancer	12 (9.6)	24 (11.7)			5 (6.4)	6 (7.7)		
Breast and reproductive system cancers	16 (12.8)	45 (21.8)			13 (16.7)	13 (16.7)		
Other cancers	7 (5.6)	36 (17.5)			6 (7.7)	13 (16.7)		
Histological grading			<0.001	0.70			0.379	0.05
I	12 (9.6)	69 (33.5)			11 (14.1)	16 (20.5)		
II	57 (45.6)	97 (47.1)			38 (48.7)	40 (51.3)		
III/undifferentiation	56 (44.8)	40 (19.4)			29 (37.2)	22 (28.2)		
AJCC Stage			<0.001	1.59			0.162	0.04
I/II	21 (16.8)	149 (72.3)			19 (24.4)	28 (35.9)		
III/IV	104 (83.2)	57 (27.7)			59 (75.6)	50 (64.1)		
Ejection fraction (%)	59.6 ± 4.8	60.5 ± 3.6	0.054	−0.27	59.9 ± 4.7	60.4 ± 3.9	0.595	0.20
Total cholesterol (μmol/L)	4.6 ± 1.2	4.7 ± 1.1	0.547	0.13	4.6 ± 1.2	4.7 ± 1.0	0.247	−0.10
High density lipoprotein (μmol/L)	1.2 ± 0.4	1.2 ± 0.3	0.080	−0.23	1.2 ± 0.4	1.2 ± 0.3	0.778	0.14
Blood urea nitrogen (μmol/L)	6.0 ± 2.5	5.8 ± 1.8	0.428	0.68	6.0 ± 2.6	5.8 ± 2.2	0.644	−0.10
Serum creatinine (μmol/L)	79.6 ± 47.5	69.5 ± 19.8	0.007	0.25	75.4 ± 30.4	72.0 ± 20.9	0.061	−0.02

PSM = propensity score matching, BMI = body mass index, AJCC = American Joint Committee on Cancer, CAD = coronary artery disease, SMD = standardized mean difference.

### 3.2 Matched patients characteristics

Characteristics of the two groups were shown in Table 1. After propensity matching, There was no difference in age, gender, BMI, alcoholism history, smoking, hypertension, diabetes mellitus, type of cancer, histological grading, tumor stage, ejection fraction, total cholesterol, high density lipoprotein, blood urea nitrogen and serum creatinine between the two matched group.

### 3.3 Analysis of association between CAD and outcome of cancer patients

**Fig. 2:**
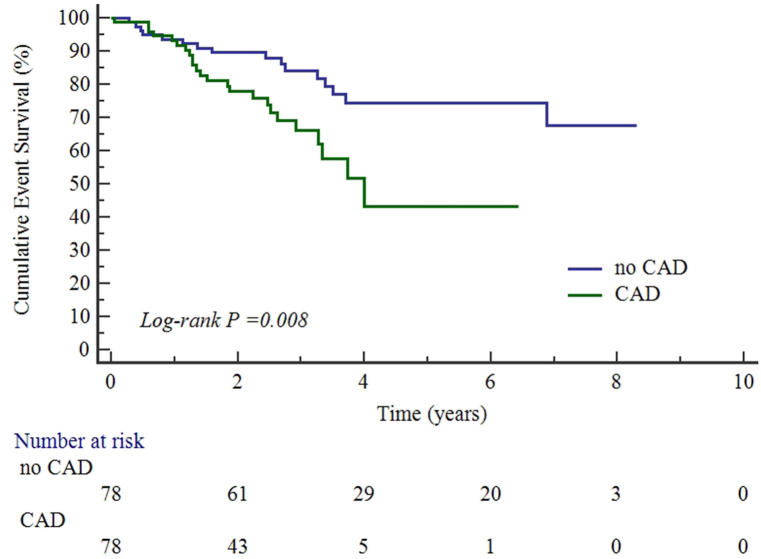
Cumulative event survival of follow-up in cancer patients with no CAD or CAD after PSM.

Five patients were lost to follow-up. After PSM, the median follow-up was 2.6 years and a total of 40 (25.6%) deaths occurred during 472 patient-years, which translates to 13.7 and 5.4 deaths per 100-patient-year in the CAD group and no CAD group, respectively. The survival free of all-cause mortality in CAD group vs. no CAD group at 1 year, 3 year and 5 years were 93.1% [*95% CI*, 87.2%–99.0%] vs. 89.7% [*95% CI,* 83.0%–96.4%] (*p* = 0.849), 61.9% [*95% CI*, 47.4%–76.4%] vs. 84.1% [*95% CI*, 75.3%–92.9%] (*p* = 0.083) and 43.1% [*95% CI*, 21.7%–64.5%] vs. 74.4% [*95% CI*, 62.4%–86.4%] (*p* = 0.009). The Kaplan-Meier curves of all-cause death were shown in Fig. 2.

### 3.4 Cox proportional hazards

On the multivariate Cox regression analysis for all-cause mortality in total patients, age (*HR*, 1.039; *95%CI*, 1.002–1.070; *p* = 0.019), smoking history (*HR*, 1.961; *95%CI*, 1.105–3.498; *p* = 0.022), alcoholism history (*HR*, 2.339; *95%CI*, 1.158–4.725; *p* = 0.018), histological grading II (*HR*, 3.627; *95%CI*, 1.010–13.024; *p* = 0.048), histological grading III/undifferentiation (*HR*, 4.135; *95%CI*, 1.089–15.699; *p* = 0.037) and CAD (*HR*, 2.444; *95%CI*, 1.251–4.775; *p* = 0.009) were significant factors for all-cause mortality. After PSM, age (*HR*, 1.040; *95%CI*, 1.001–1.081; *p* = 0.014) and CAD (*HR*, 2.164; *95%CI*, 1.057–4.430; *p* = 0.035) remained significant factors for all-cause mortality (Table 2).

**Table 2:** Factors associated with overall survival of the patients before and after PSM.

Characteristic	Before PSM				After PSM			
	Univariate analysis		Multivariate analysis		Univariate analysis		Multivariate analysis	
	HR (95% CI)	p value	HR (95% CI)	p value	HR (95% CI)	p value	HR (95% CI)	p value
Age (years)	1.068 (1.039–1.097)	<0.001	1.039 (1.002–1.070)	0.019	1.075 (1.033–1.119)	<0.001	1.040 (1.001–1.081)	0.014
Gender (male)	2.383 (1.443–3.934)	0.001	0.755 (0.376–1.518)	0.430	1.785 (0.942–3.383)	0.075	2.515 (0.935–6.765)	0.068
BMI (kg/m^2^)	0.932 (0.869–0.999)	0.047	0.977 (0.907–1.052)	0.534	0.890 (0.807–0.982)	0.020	0.901 (0.787–1.031)	0.130
History of smoking	3.417 (2.110–5.533)	<0.001	1.961 (1.105–3.498)	0.022	1.263 (0.527–3.028)	0.600	0.186 (0.022–1.600)	0.126
History of alcoholism	4.502 (2.605–7.780)	<0.001	2.339 (1.158–4.725)	0.018	2.013 (0.785–5.164)	0.146	3.526 (0.369–33.739)	0.274
Hypertension	1.712 (1.053–2.784)	0.030	1.143 (0.643–2.035)	0.649	1.110 (0.596–2.067)	0.741	1.514 (0.694–3.303)	0.298
Diabetes mellitus	2.481 (1.527–4.029)	<0.001	1.490 (0.860–2.582)	0.154	1.709 (0.869–3.363)	0.121	1.196 (0.567–2.526)	0.638
Histological grading								
I	ref		ref		ref		ref	
II	7.330 (2.236–24.029)	0.001	3.627 (1.010–13.024)	0.048	4.564 (1.069–19.488)	0.040	1.489 (0.202–10.987)	0.696
III/undifferentiation	10.961 (3.278–36.651)	<0.001	4.135 (1.089–15.699)	0.037	4.759 (1.099–20.605)	0.037	0.975 (0.115–8.281)	0.982
AJCC Stage								
I/II	ref		ref		ref		ref	
III/IV	4.470 (2.575–7.760)	<0.001	1.280 (0.603–2.715)	0.520	1.774 (0.842–3.736)	0.132	1.734 (0.613–4.901)	0.299
Ejection fraction (%)	0.950 (0.903–1.000)	0.052	0.996 (0.943–1.051)	0.872	0.915 (0.855–0.980)	0.011	0.949 (0.873–1.033)	0.225
Total cholesterol (μmol/L)	0.717 (0.568–0.904)	0.005	0.896 (0.693–1.159)	0.403	0.739 (0.535–1.020)	0.066	1.016 (0.718–1.437)	0.930
High density lipoprotein (μmol/L)	0.417 (0.191–0.911)	0.028	0.727 (0.300–1.759)	0.479	0.332 (0.107–1.027)	0.056	0.237 (0.053–1.062)	0.060
Blood urea nitrogen (μmol/L)	0.998 (0.893–1.114)	0.967	1.005 (0.881–1.146)	0.945	1.033 (0.923–1.157)	0.570	0.977 (0.848–1.124)	0.742
Serum creatinine (μmol/L)	1.002 (0.996–1.009)	0.497	0.994 (0.981–1.007)	0.397	0.999 (0.987–1.012)	0.903	0.991 (0.973–1.010)	0.374
CAD	5.235 (3.142–8.720)	<0.001	2.444 (1.251–4.775)	0.009	2.386 (1.238–4.597)	0.009	2.164 (1.057–4.430)	0.035

PSM = propensity score matching, HR = hazard ratios, CI = confidence interval, BMI = body mass index, AJCC = American Joint Committee on Cancer, CAD = coronary artery disease.

## 4 Discussion

The present study revealed that CAD assessed by coronary CTA was an independent predictor of all-cause mortality in cancer patients without a history of CVD. Even if the cancer can be treated surgically, patients with cancer are potentially at higher risk for CVD and mortality compared those without cancer [[18], [19], [20]]. The CAD status assessed by coronary CTA could add prognostic value and be a key consideration for preventing adverse events in these patients.

CAD assessed by coronary CTA was seen in 38.8% of cancer patients with angina symptoms. It is appreciated that CAD and cancer share associated risk factors of male gender, age and smoking [20]. After PSM, age was risk factor for all-cause mortality in multivariate Cox regression analysis, which was consistent with the growing evidence [21,22].

Invasive coronary angiography (ICA) was initially used as a tool of quantification of CAD scoring systems for both clinical practice and scientific investigation [23]. Coronary CTA is a relatively new test that enables noninvasive and direct visualization of the presence and extent of coronary stenosis [12,24]. Increasing amounts of CAD scoring based on coronary CTA including coronary artery calcification (CAC) are used in clinical and research [[25], [26], [27], [28]]. Cardiovascular disease is a competing cause of mortality in the early-stage of cancer [29]. With the rise of cardio-oncology, accumulating researches focused on the prevention and early detection of cardiovascular disease in patients treated for cancer [30]. Coronary CTA is considered to change the landscape of coronary assessment in the field of cardiology. However, its role in cardio-oncology is primarily restricted to assessment of coronary calcium and CAD [31].

A recent oncology study revealed that the present of CAC on the CT portion of FDG/PET-CT was associated with all-cause mortality and cardiac events on univariable and multivariable analysis in a median follow up of 41 months [32]. Similarly, the present of CAC at chest CT was demonstrated to predictive of all-cause mortality and cardiac events in breast cancer patients [33]. To date, the CAC or CACS (coronary artery calcification score) has been considered a reliable index for cardiovascular risk assessments in asymptomatic adults. However, the main limitation of these tools is the lack of data for the management or downstream testing strategies according to CAC or CACS [34]. Although it is clear that an absence of CAC guarantees a very good prognosis in a long-term follow-up, no consensus exists for the downstream screening strategies or treatment of patients with the present of CAC [35]. Compared with CAC, which is widely used for all-cause mortality and major adverse cardiovascular events (MACEs) prediction in asymptomatic individuals, coronary CTA is able to further assess coronary plaque burdens and characteristics. The current guideline or consensus do not yet establish whether CAD screening with coronary CTA should be performed in cancer patients. In the present study, CAD was associated with adverse outcome in cancer patients using propensity matching method. Although receiving optimal medical therapy and percutaneous coronary intervention, poor prognosis was found in cancer patients with CAD. Previous study showed that 26%, 22% of one-year estimated survival rate was reported in cancer patients with non-ST elevation myocardial infarction and ST elevation myocardial infarction, respectively [36]. In particular, CAD in cancer patients does not often caused by the toxicity of cancer therapy, and it may be associated with aging or the deterioration of potential cardiovascular disease. Therefore, the identification and management of CAD in cancer patients in early stage are critical to preserve the survival benefits of modern cancer therapy [37].

Our study had several limitations. First, this study was retrospective study with potential for selection bias and missing values. To overcome these limitations, we performed Cox multivariate analysis and propensity-matching analysis with adjustment for potential confounders, but could not adjust for unmeasured potential confounders. Second, there was lack of on how long between CAD diagnosis and cancer diagnosis. Third, the follow-up duration was relatively short to assess the long-term all-cause mortality and the present study lack the cause of death. Forth, this study did not include the intervening variables and the composition of the sample in the final analysis as these information was unavailable. Due to the unavailability of the data on specific causes of death, the clinical endpoint was all-cause death. Cardiovascular death could not be separately assessed as an additional outcome which would be expected to have a stronger correlation with the presence of CAD. In addition, the present study did not consider the intervening variables and limited it to the specific population diagnosed with cancer indicates the presence of biases, which limit the research findings. Fifth, as this is a single center retrospective study, due to lack of treatment information, the information on the number of patients receiving cancer therapy and the number of patients in complete remission was unable to provide in the present study. Finally, the present study includes a relatively small sample size that was unable for more grouping. Further studies should include more patients to improve the classification scheme. Nevertheless, this study suggests that coronary CTA will be instrumental in risk stratification of cancer patients. Further studies are needed to better define these observations.

In summary, in our one-center and small sample study, compared to no CAD evaluated by coronary CTA, CAD increased the all-cause mortality of cancer patients and this effect remains significant after adjusting confounders. Due to the retrospective design and lack of information on some medical history and treatments, especially immune checkpoint inhibitors, a large-scale prospective study is needed to further determine the prognostic value of coronary CTA in cancer patients.

## Ethics statement

This study has been approved by the Ethics Committee of the Central Hospital of Wuhan, Tongji Medical College, Huazhong University of Science and Technology (Code: WHZXKYL2022-140).

## Funding and acknowledgments

No funding support.

## Data availability statement

Data will be made available when requested.

## CRediT authorship contribution statement

**Zengfa Huang:** Writing – review & editing, Writing – original draft, Formal analysis. **Tao Li:** Writing – original draft, Formal analysis. **Shan Zhang:** Writing – original draft. **Bei Jiang:** Writing – original draft. **Mei Li:** Supervision, Methodology, Data curation. **Beibei Cao:** Supervision, Methodology, Data curation. **Hongfeng Zhang:** Data curation. **Zhiqiang Zhou:** Methodology, Conceptualization. **Zheng Huang:** Methodology, Conceptualization. **Xiang Wang:** Writing – review & editing, Conceptualization.

## Declaration of competing interest

The authors declare that they have no known competing financial interests or personal relationships that could have appeared to influence the work reported in this paper.
